# Indoleamine-2,3-dioxygenase activity induces neutrophil apoptosis

**DOI:** 10.1186/cc9628

**Published:** 2011-03-11

**Authors:** K Van der Sluijs, R Singh, A Dijkhuis, M Snoek, R Lutter

**Affiliations:** 1 Academic Medical Center, Amsterdam, the Netherlands

## Introduction

Influenza-related mortality is often caused by secondary bacterial pneumonia. We have previously shown that the tryptophan-catabolizing enzyme indoleamine-2,3-dioxygenase (IDO) critically impairs host defense against secondary bacterial pneumonia [1]. Since inhibition of IDO resulted in increased neutrophil numbers during primary viral infection, we hypothesized that tryptophan degradation and/or the generation of downstream metabolites induces neutrophil apoptosis. In the present study we aimed to investigate the impact of IDO-mediated tryptophan metabolism on neutrophil apoptosis *in vitro *and in *vivo*.

## Methods

Freshly isolated neutrophils were cultured in the presence or absence of tryptophan, kynurenine and 3-hydroxy-anthranilic acid. Apoptosis was identified by annexin V/propidium iodine staining (%, mean ± SD). To confirm our *in vitro *data, transgenic mice that conditionally express IDO in the airway epithelium upon doxycycline (dox) treatment and control mice were challenged with LPS (1 μg) or *Klebsiella pneumoniae *(10^4 ^colony-forming units) intranasally and sacrificed after 24 hours to count neutrophils in bronchoalveolar lavage fluid (total number, mean ± SD). Statistical analysis was performed by Student's *t *test or Mann-Whitney U test where appropriate. *P *< 0.05 was considered significant.

## Results

Both kynurenine and 3-hydroxy-anthranilic acid enhanced apoptosis in freshly isolated neutrophils (60.3 ± 8.7% and 45.5 ± 1.7% respectively vs. 33.5 ± 8.1% under control conditions, both *P *< 0.05), which was reversed by adding tryptophan. Conditional transgenic mice, which showed marked expression of IDO in the pulmonary compartment, had reduced neutrophil numbers in bronchoalveolar lavage fluid after challenge with *K. pneumoniae *(3.36 ± 1.92 × 10^5 ^vs. 12.1 ± 9.0 × 10^5 ^in dox-treated littermates, *P *< 0.05) and LPS (1.88 ± 1.22 × 10^5 ^vs. 5.21 ± 3.81 × 10^5 ^in control-treated transgenic mice, *P *< 0.05), which was associated with active caspase-3 staining in dox-treated mice, but not in control mice.

## Conclusions

Neutrophils undergo apoptosis in presence of kynurenine or 3-hydroxy-anthranilic acid and the absence of tryptophan. Pulmonary IDO expression, as occurs during influenza infection, enhances neutrophil apoptosis *in vivo *and may impair host defense against secondary bacterial infections.
